# Preliminary Findings on Proline-Rich Protein 14 as a Diagnostic Biomarker for Parkinson’s Disease

**DOI:** 10.1007/s12017-020-08617-z

**Published:** 2020-10-01

**Authors:** Tao Jin, Xuling Tan, Xiaoliu Shi, Lingling Lv, Xinke Peng, Hainan Zhang, Beisha Tang, Chunyu Wang, Mei Yang

**Affiliations:** 1 Department of Medical Genetics, The Second Xiangya Hospital, Central South University, 139 Middle Renmin Road, Changsha, 410011 Hunan China; 2 Center for Medical Genetics, School of Life Sciences, Central South University, Changsha, 410008 Hunan China; 3 Department of Neurology, The Second Xiangya Hospital, Central South University, 139 Middle Renmin Road, Changsha, 410011 Hunan China; 4 Department of Neurology, Xiangya Hospital, Central South University, Changsha, 410008 Hunan China; 5 Department of Orthopedics, The Second Xiangya Hospital, Central South University, 139 Middle Renmin Road, Changsha, 410011 Hunan China; 6 Hunan Key Laboratory of Tumor Models and Individualized Medicine, The Second Xiangya Hospital, Central South University, Changsha, China

**Keywords:** Parkinson’s disease, PRR14, Biomarker, Serum

## Abstract

The nuclear envelope component proline-rich protein 14 (PRR14) is involved in the nuclear morphological alteration and activation of the mTOR (mammalian target of rapamycin) signaling pathway, and has been repeatedly shown to be upregulated in patients with Parkinson’s disease (PD). The aim of this study was to explore whether PRR14 can be used as a potential biomarker for the diagnosis of PD. We compared PRR14 expression in PD patients and normal controls in gene expression omnibus (GEO) data. Quantitative enzyme-linked immunosorbent assay (ELISA) was used to detect PRR14 expression in PD patients and age- and sex-matched controls. The relationship between serum PRR14 and clinical phenotype was evaluated using correlation analysis and logistic regression. The expression of PRR14 in whole blood, substantia nigra, and medial substantia nigra was significantly higher in PD patients than in the healthy control group. Compared to plasma, serum was more suitable for the detection of PRR14. Furthermore, serum PRR14 level in PD patients was significantly higher than that in age- and sex-matched controls. The area under the curve for serum PRR14 level in the ability to identify PD versus age- and sex-matched controls was 0.786. In addition, serum PRR14 level was found to correlate with constipation in PD patients. Our findings demonstrate for the first time that serum PRR14 is a potential biomarker for PD.

**Electronic supplementary material:**

The online version of this article (10.1007/s12017-020-08617-z) contains supplementary material, which is available to authorized users.

## Background

The diagnosis of Parkinson’s disease (PD), the second most common neurodegenerative disease, is still difficult due to the large symptom overlap of PD and multiple system atrophy, Lewy body dementia, and corticobasal ganglionic degeneration (Pringsheim et al. 2014; Dorsey and Bloem 2018; Ascherio and Schwarzschild 2016; Tolosa et al. 2006). Therefore, the identification of reliable biomarkers is of great significance for revealing its pathogenesis, improving the accuracy of diagnosis, monitoring disease progression, and assessing drug treatment outcomes.

The cell nucleus plays a central role in cell’s function, and nuclear architecture defects have been shown to correlate with the manifestation of aging and a number of age-related human diseases, such as cancer(Zink et al. 2004), Hutchinson–Gilford progeria syndrome (Eriksson et al. 2003), and PD itself (Liu et al. 2012). Similarly, the dysregulation of mTOR signaling pathway plays an important role in aging and age-related diseases, the inhibition of which slows down the process of aging and protects neurons in PD (Johnson et al. (2013; Tain et al. 2009). The nuclear envelope component proline-rich protein 14 (PRR14) physically and functionally tethers nuclear lamina and heterochromatin together, and is able to regulate nuclear morphology (Poleshko et al. 2013; Yang and Yuan 2015) and activate the mTOR signaling pathway (Yang et al. 2016). In addition, the upregulation of PRR14 is repeatedly detected in PD patients and animal models of PD (Soreq et al. (2012); Sinha et al. 2009). Hence, PRR14 may be involved in the pathogenesis of PD.

In this study, we investigated whether PRR14 may serve as a biomarker for PD in patients and its relationship with clinical symptoms of PD.

## Methods

### Participants

This study included 108 patients with sporadic PD and 50 age- and sex-matched normal controls (NC) without objective cognitive impairment or Parkinson’s disease symptoms. The subjects were enrolled at the Department of Neurology, Second Xiangya Hospital, Central South University. All of the participants were administered neurological and cognitive assessments by medical doctors. PD patients were clinically diagnosed according to the MDS Clinical Diagnostic Criteria for PD (Postuma et al. 2015). Patients who were taking anti-inflammatory medication or had been diagnosed with other neurodegenerative diseases, tumors, coronary heart disease, diabetes mellitus, or autoimmune diseases were excluded from the study.

### Clinical Evaluation

We collected information including age, sex, duration of illness, education, and medication. Levodopa equivalent dose (LED) was calculated as described in previous studies (Wang et al. 2006). To evaluate the status of PD patients, Unified Parkinson’s disease Rating Scale (UPDRS), Hoehn and Yahr stages (H–Y stages), Non-motor Symptoms Scale (NMSS), Mini-mental State Examination (MMSE), Parkinson's disease Sleep Scale (PDSS), REM Sleep Behavior Disorder Questionnaire-Hong Kong (RBDQ-HK), Epworth Sleepiness Scale (ESS), 6-item Hyposmia Rating Scale (HRS), Hamilton Depression Scale (HAMD), and Parkinson's disease Quality of Life Questionnaire (PDQ39 scores) were assessed in PD patients and were consequently categorized based on the median or previously reported thresholds (Chen et al. 2015; Shen et al. 2014; Simuni et al. 2015; Zhou et al. 2014; Martinez-Martin et al. 2016; Zuo et al. 2016; Nasreddine et al. 2010).

### Sample Collection and Measurement of Biochemical Indicators

Venous blood from participants was centrifuged at a speed of 200 g for 10 min at 4 °C, and serum was collected and stored at − 80 °C. The level of PRR14 in blood or serum was determined by PRR14 enzyme-linked immunosorbent assay (ELISA) kit (Jianglai Bio, China). The minimum detectable dose of PRR14 was 2.5 ng/mL, and the range of the kit was from 0.125 ng/well to 4 ng/well.

Based on the median value of PRR14, PD patients were divided into a High PRR14 group and a Low PRR14 group.

### Data Analysis

All of the data were statistically analyzed using SPSS22.0 (IBM Corp, Armonk, New York). For continuous variables, the Kolmogorov–Smirnov test was utilized to judge the normality of the sample distribution. Normally distributed data were represented as mean ± standard deviation (SD), whereas non-normal data were presented as median (interquartile range). We further used the Levene’s test for the homogeneity of continuous normally distributed data. Homogeneous data underwent independent sample *t*-tests for comparison between groups, whereas non-homogeneous data underwent *t*-tests with correction. Continuous variables that did not show normal distribution were compared using the Mann–Whitney *U* test. Correlation analysis was performed using the Pearson and Spearman test. Categorical variables were presented as percentage (%), and the comparison between groups was analyzed by the Pearson chi-square and Fisher’s exact tests. We also computed receiver operating characteristic curves (ROCs) to determine sensitivity and specificity. Finally, we used logistic regression to analyze the relationship between PRR14 level and clinical features. All of the tests were set at *P* < 0.05 for statistical significance.

## Results

### PRR14 is Upregulated in PD Patients

The gene expression profiles were screened in the gene expression omnibus (GEO) database. The expression data of PRR14 in the whole blood sample (*P* = 0.003, Online Resource Fig. a) (Scherzer et al. 2007), substantia nigra (*P* = 0.001, Online Resource Fig. b) (Lesnick et al. 2007), and medial substantia nigra (*P* = 0.001, Online Resource Fig. c) (Moran et al. 2006) from PD patients and the normal controls (NC) were extracted from the GEO database. Analysis showed that PRR14 was significantly upregulated in all three regions in PD patients. However, the superior frontal gyrus, which is a brain region not directly related to PD, did not show any difference (Online Resource Fig. c).

### The Expression of PRR14 in Serum and Plasma

**Fig. 1:**
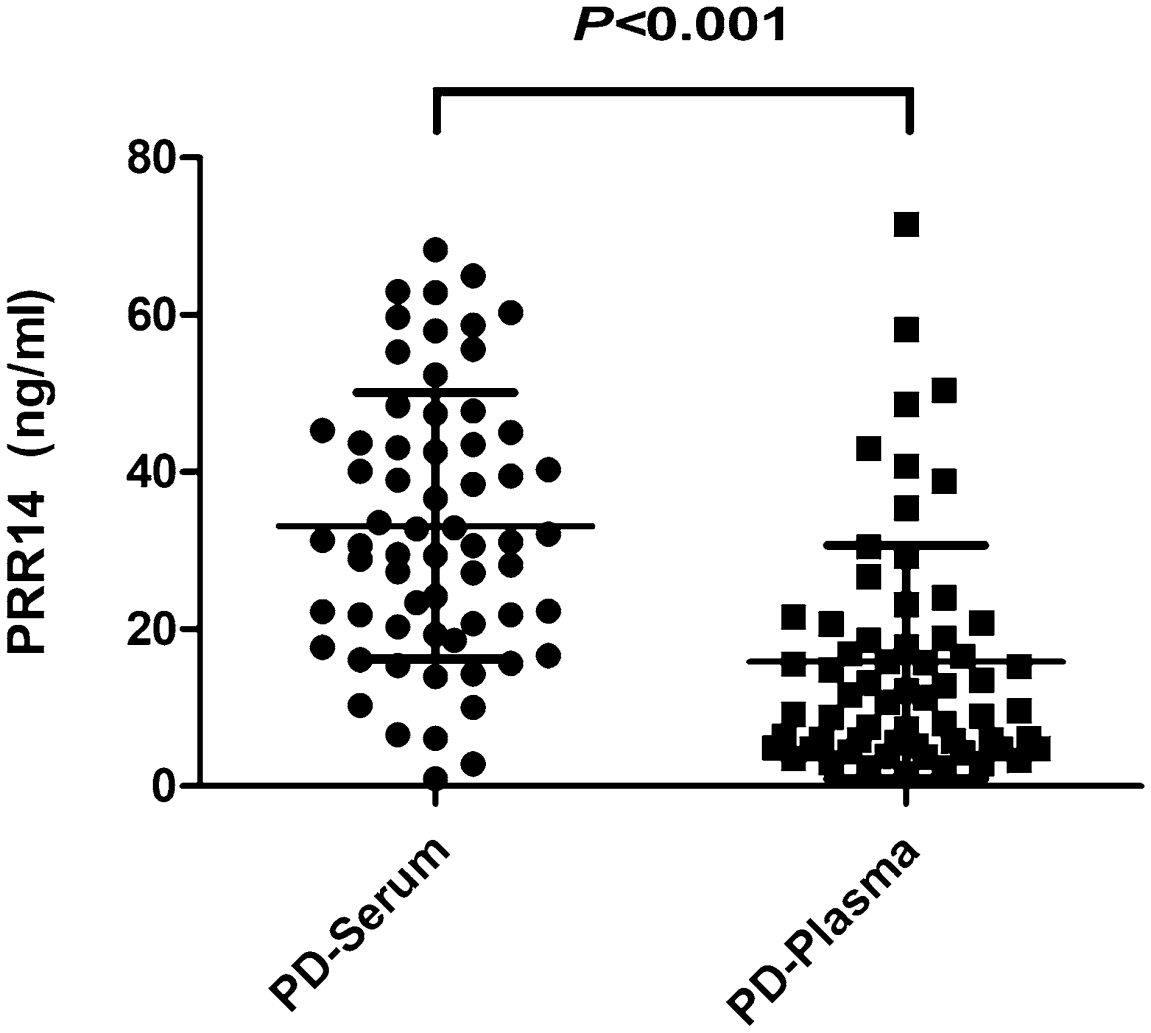
The expression of PRR14 in serum and plasma from PD patients. The levels of PRR14 in serum and plasma from the same cohort of PD patients were quantified by ELISA and statistically analyzed by paired two-tailed Student’s *t*-tests

Serum and plasma were randomly selected from 63 PD patients, and the level of PRR14 in serum and plasma was detected by an ELISA kit. The paired *t*-test showed that the levels of PRR14 in PD patients’ serum and plasma were significantly different (*P* < 0.001, Fig. 1). A Kolmogorov–Smirnov normality test showed that the expression of PRR14 in serum from PD patients was normally distributed (*P* = 0.200), while that in plasma was not (*P* < 0.001). Since the statistical inference method in large samples is based on their normal distribution, serum is more suitable than plasma to detect the expression of PRR14. We therefore selected serum for the subsequent detection of PRR14.

### Serum PRR14 is Upregulated in PD Patients

**Fig. 2:**
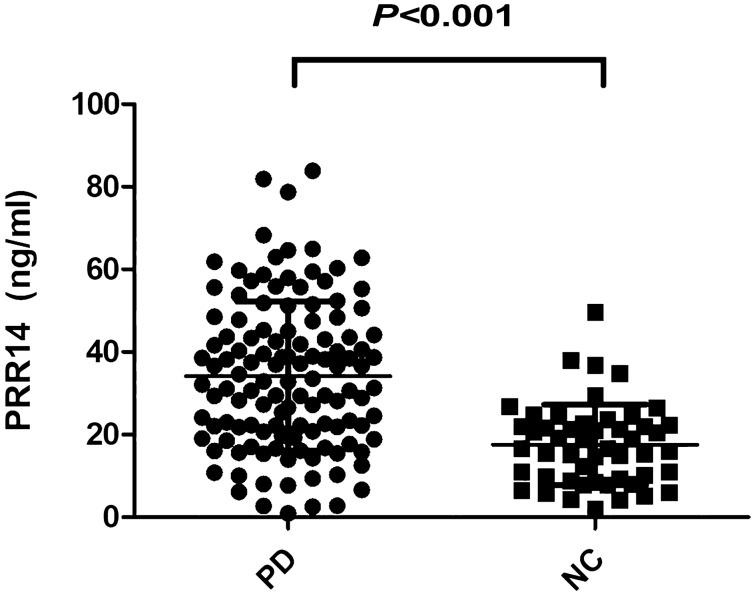
Serum PRR14 in PD patients and NC. Serum PRR14 in PD patients and NC was quantified by ELISA and statistically analyzed by unpaired two-tailed Student’s *t*-test

A total of 158 participants were included in the study, including PD patients (*n* = 108) and NC (*n* = 50). Relevant information of the participants is shown in Table 1. Among the PD patients, 48 were in H–Y stages 1–1.5; 52 were in H–Y stages 2–2.5; and 6 were in H–Y stages 3–5. We observed significant differences in serum PRR14 between the PD (34.2 ± 18.0 mg/mL) and NC groups (17.5 ± 9.8 mg/mL) (*P* < 0.001) (Fig. 2).

**Table 1:** Demographic data and serum PRR14 in the PD and NC groups

Items information	PD (*n* = 108)	NC (*n* = 50)	*P* value
Male [*n* (%)]^a^	52 (48.1)	25 (50)	0.829
Age (years)^b^	60.5 (52.2–67.0)	56.0 (55.0–59.2)	0.115
H–Y stage (mean ± SD)	1.9 ± 0.6	–	–
Duration (year, mean ± SD)	2.0 (1.0–4.0)	–	–
LED (mean ± SD)	412.5 (259.3–450.0)	–	–
PRR14^c^	34.2 ± 18.0	17.5 ± 9.8	< 0.001

*H–Y* Hoehn and Yahr, *LED* levodopa equivalent dose, *SD* standard deviation

Age, duration and LED do not conform to normal distribution, presented by median and quartile

^a^Chi-square test

^b^Mann–Whitney *U* test

^c^Student t-test

**Fig. 3:**
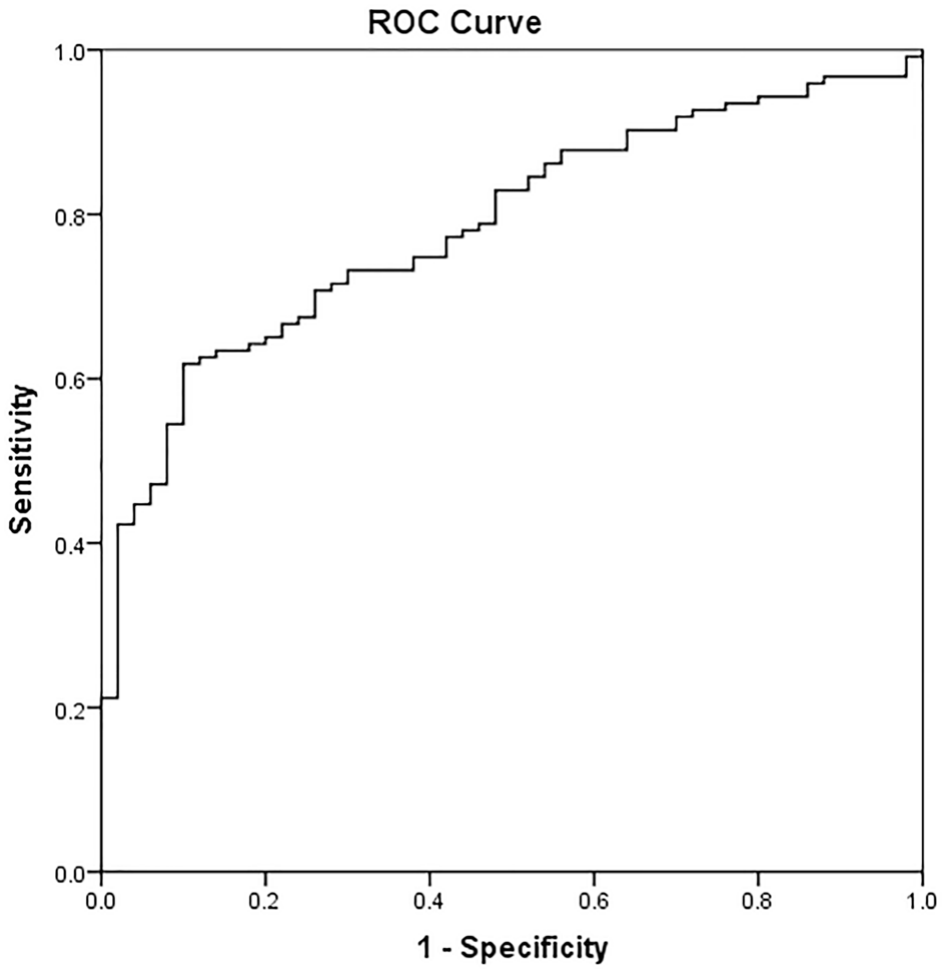
ROC curve of PRR14 as biomarker for PD

The ROC statistic denoting the ability to differentiate PD patients from NC using PRR14 was 0.786 (95% CI 0.717–0.854) and the cutoff value was 27.0 ng/mL. Sensitivity and specificity were 61.8% and 90.0%, respectively (Fig. 3).

We further examined the association between serum PRR14 level and clinical symptoms. No significant correlation between serum PRR14 was found with any of the rated clinical symptoms, which were assessed by UPDRS, UPDRS-2, UPDRS-3, NMSS, NMSE, PDSS, RBDQ-HK, ESS, HRS, HAMD, PDQ39 or duration of the disease (Table 2). For symptoms not rated, we compared their prevalence in PD patients divided into the High-PRR14 and Low-PRR14 groups using a Pearson chi-square test. This method still did not yield any significant differences between groups in symptoms including severe daily activities impairment, severe motor impairment, wearing-off, rapid eye movement sleep behavior disorder (RBD), excessive daytime sleepiness (EDS), constipation, hyposmia, depression, sleep disturbance, abnormal involuntary movements (AIMs), freezing of gait (FOG), or dementia. However, after controlling for age, sex, and duration of disease, logistic regression analysis showed that the risk of constipation in the high PRR14 group was 2.403 times higher than that in the low PRR14 group (95% CI 0.989–5.840, *P* = 0.029), as shown in Table 3.

**Table 2:** Association between serum PRR14 level and clinical phenotype

	r	*P* value
UPDRS-2	0.068	0.487
UPDRS-3	− 0.029	0.771
UPDRS*	− 0.013	0.894
NMSS	0.083	0.396
MMSE	− 0.059	0.550
PDSS	0.001	0.990
RBDQ-HK	0.057	0.559
ESS	− 0.060	0.546
HRS	− 0.019	0.843
HAMD	0.072	0.460
PDQ39	0.024	0.809
Duration	− 0.104	0.295

*Pearson correlation analysis; The rest was analyzed by Spearman correlation

**Table 3:** Association between high serum PRR14 and clinical phenotype

	Low PRR14	High PRR14	OR (95% CI)	*P*	Adjusted *P* value
Severe daily activities impairment (%)	41.5	39.6	0.925 (0.426–2.008)	0.843	0.351^b^
Severe motor impairment (%)	28.3	35.8	1.416 (0.624–3.214)	0.406	0.516^b^
Wearing-off (%)	3.8	5.8	1.531 (0.245–9.561)	0.649	0.568^a^
RBD (%)	26.4	26.4	1.000 (0.422–2.372)	1.000	0.750^a^
EDS (%)	42.3	35.8	0.762 (0.347–1.672)	0.498	0.481^a^
Constipation (%)	18.9	35.8	2.403 (0.989–5.84)	0.053	0.029^a^
Hyposmia (%)	24.5	37.7	1.865 (0.808–4.305)	0.144	0.370^a^
Depression (%)	24.5	28.3	1.215 (0.511–2.885)	0.660	0.459^a^
Sleep disturbance (%)	7.5	5.7	0.735 (0.156–3.456)	0.697	0.739^a^
AIMs (%)	7.5	5.7	0.735 (0.156–3.456)	0.697	0.791^b^
FOG (%)	7.5	5.7	0.735 (0.156–3.456)	0.697	0.989^b^
Dementia (%)	13.2	11.5	0.857 (0.267–2.747)	0.795	0.661^b^

^a^Adjusted for gender, age, and duration of disease

^b^Adjusted for gender, age, duration, and LED

## Discussion

PRR14 has been demonstrated to be one of the most upregulated proteins in PD patients’ cerebrospinal fluid (Sinha et al. 2009), as well as in whole blood samples (Scherzer et al. 2007), substantia nigra (Lesnick et al. 2007), and medial substantia nigra (Moran et al. 2006) (Online Resource Figure). Similar upregulation has also been detected in MPTP-treated mouse model (Soreq et al. 2012). Our study is consistent with these findings, showing that PRR14 is increased in PD patients and establishes its potential as a biomarker for PD. Moreover, it appears that serum PRR14 has high specificity (90.0%) but lower sensitivity (61.8%).

Although PD is clinically defined by its motor symptoms, non-motor symptoms (NMS) are common in PD patients, and these symptoms may appear in both early pre-symptomatic stages and throughout the disease course. Recently, there has been more focus on gastrointestinal dysfunction in PD, and current research tends to agree on the fact that PD derives from pathogens passing through defects in intestinal epithelial barrier into the intestinal neurons (Braak et al. 2006; Forsyth et al. 2011; Clairembault et al. 2015; Olanow and Prusiner 2009). Constipation, which reflects gastrointestinal dysfunction, is one of the most common NMS in PD (Poewe et al. 2017; Quigley 1996). Consistent with this assumption, constipation has been reported in PD patients as early as 20 years before the onset of motor symptoms (Savica et al. 2009), and individuals with severe constipation history have a higher risk of developing PD in future (Savica et al. 2009; Abbott et al. 2001). Our results revealed that serum PRR14 is only associated with constipation after controlling for age, sex, and duration of disease (*P* = 0.029), suggesting that PRR14 may play a role in the pathogenesis of PD. Such a finding would explain the lack of association between serum PRR14 and other clinical symptoms.

In previous studies, PRR14 was identified as a strong activator of the mTOR signaling pathway (Yang et al. 2016; Ren et al. 2020). If it similarly regulates the mTOR signaling pathway in PD patients, this would indicate that PRR14 contributes to PD pathogenesis via dysregulation of the mTOR signaling pathway, thus protecting dopaminergic neurons from loss (Segarra et al. 2006). Further studies are needed to explore PRR14′s function in PD in greater detail.

This study has some limitations. First, the sample size is rather small and serum PRR14 is not detected in other neurodegenerative diseases. Therefore, future studies may wish to increase sample size and expand sample range to gain a fuller understanding of the mechanisms of PRR14. Second, most PD patients in the study were on a regimen of PD drugs, the effect of which may have weakened the correlation between serum PRR14 and clinical symptoms, inviting the need for future studies that examine the effects of the serum in medication-naïve patients.

## Conclusions

In conclusion, we have found evidence that serum PRR14 is significantly higher in PD patients compared to normal controls. Furthermore, we found that PRR14 is associated with constipation. These results indicate that PRR14 may be a novel biomarker of PD.

## Electronic supplementary material

Below is the link to the electronic supplementary material.

Supplementary file1 (DOCX 201 kb)

## Abbreviations

PD: Parkinson’s disease
NC: Normal controls
SD: Standard deviation
LED: Levodopa equivalent dose
UPDRS: Unified Parkinson’s disease Rating Scale
H-Y stages: Hoehn and Yahr stages
NMSS: Non-motor Symptoms Scale
MMSE: Mini-mental State Examination
PDSS: Parkinson's disease Sleep Scale
RBDQ-HK: REM Sleep Behavior Disorder Questionnaire - Hong Kong
ESS: Epworth Sleepiness Scale
HRS: 6-Item Hyposmia Rating Scale
HAMD: Hamilton Depression Scale
PDQ39: Parkinson's disease Quality of Life Questionnaire
ELISA: Enzyme-linked immunosorbent assay
GEO: Gene expression omnibus
GSE: GEO series
RBD: Rapid eye movement sleep behavior disorder
ROC curve: Receiver operating characteristic curve
EDS: Excessive daytime sleepiness
AIMs: Abnormal involuntary movements
FOG: Freezing of gait
