# Supplementary material for: Preliminary Findings on Proline-Rich Protein 14 as a Diagnostic Biomarker for Parkinson’s Disease
Source: Neuromolecular Med. 2020 Oct 1;23(2):285–91. doi: 10.1007/s12017-020-08617-z (PMC8128746; doi:10.1007/s12017-020-08617-z)
Supplement: Supplementary file 1 — Supplementary file1 (DOCX 201 kb) [file 12017_2020_8617_MOESM1_ESM.docx]

**Supplemental Materials:**

The gene expression profiles were screened in the gene expression omnibus (GEO) database. Criteria for selecting the data sets were as follows: (1) samples from PD patients and NC are included; (2) the number of samples is greater than 20; (3) studies which comprised of CEL raw files. Finally, three GEO series (GSE) profiled by array were employed in our study, which were GSE6613, GSE7621 and GSE8397, respectively. GSE6613 included whole blood expression data from 50 PD patients, 33 patients with neurodegenerative diseases other than PD, and 23 NC. GSE7621 included substantia nigra expression profiles from 9 NC and 16 PD patients. And 47 individual tissue samples from substantia nigra of PD patients and were profiled in GSE8397.


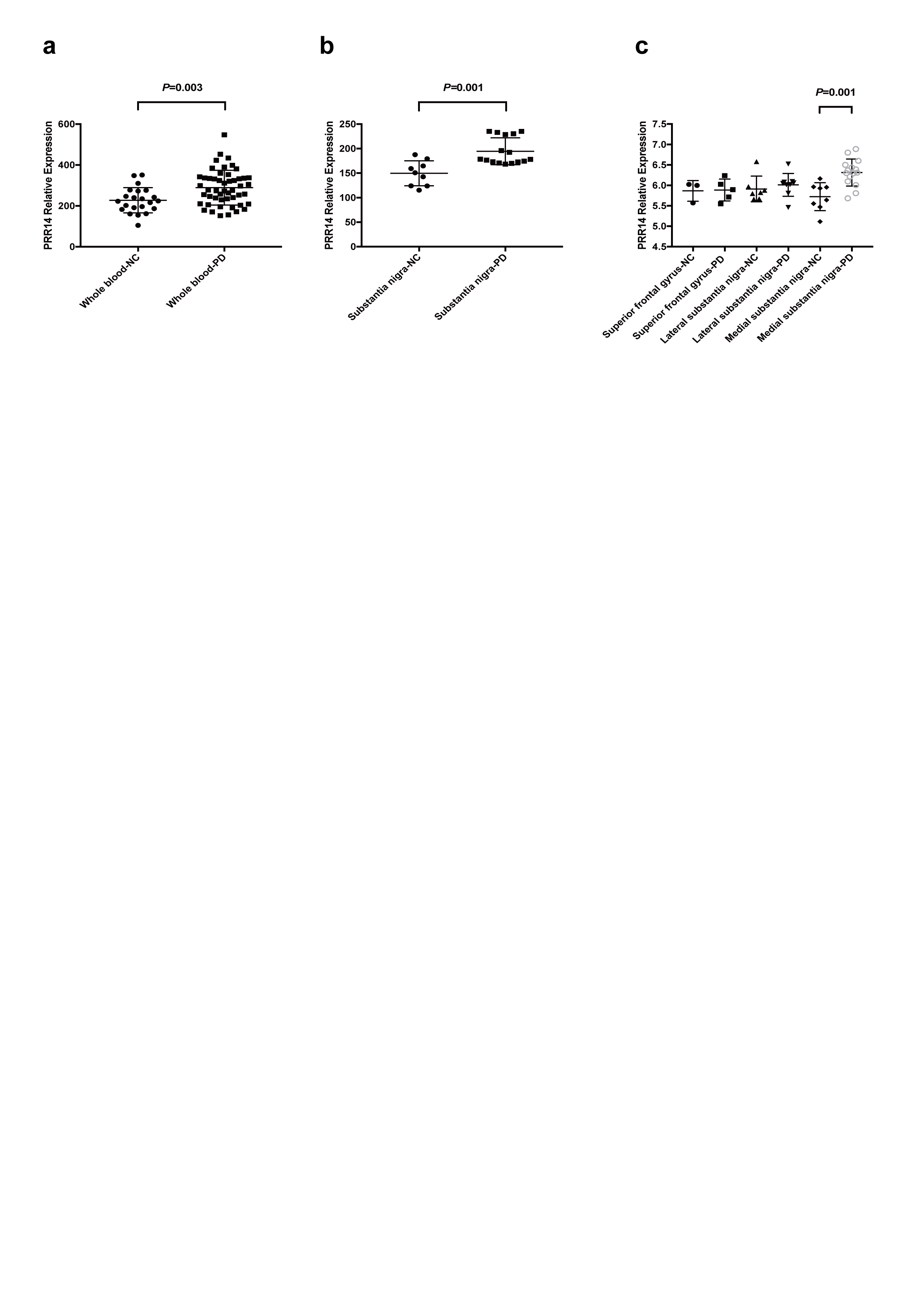
**Online Resource Fig:** The expression of PRR14 in PD patients. The transcription data of PRR14 in whole blood (a), substantia nigra (b) and specific brain areas (c) from PD patients and NC, quantified by array, were extracted from GEO database. Unpaired Student's t-test was performed.
